# Descriptive attributes used in the characterization of stingless bees (Apidae: Meliponini) in rural populations of the Atlantic forest (Misiones-Argentina)

**DOI:** 10.1186/1746-4269-8-9

**Published:** 2012-02-13

**Authors:** Fernando Zamudio, Norma I Hilgert

**Affiliations:** 1 Instituto de Biología Subtropical. Facultad de Ciencias Forestales, Universidad Nacional de Misiones, CONICET, Andresito 21, 3370 Puerto Iguazú, Misiones, Argentina

**Keywords:** Ethnozoology, *Criollos*, Cognitive prototypes, *Emic*, Descriptive traits, Salient criteria

## Abstract

**Abstract:**

**Background**

Human beings employ a combination of morphological, sensorial, utilitarian, cultural and ecological characters when they identify and classify organisms. Ethnotaxonomy has provided a store of information about the characters cultures employ when they identify and classify a vast diversity of taxonomic groups. Nevertheless, some more research is needed to provide a comparison of the characters employed in the description of taxons, and an analysis of the extent to which those descriptors are represented. Stingless bees constitute a diverse group of social insects that have been widely studied from an ethnobiological perspective due to their utilitarian and cultural importance. The objective of this study is to identify the elements local people consider when characterizing stingless bees, and how important these elements are in the study of local classifications.

**Methods**

The methodology used involves semi-structured interviews and trips with the informants to rural areas. Locally known ethnospecies are characterized, descriptive traits and salient criteria used in those characterizations are identified, and the frequency of reference of descriptive traits and salient criteria are estimated. Besides, the descriptive traits used for each ethnospecies are compared, and the contribution of the characterizations as a heuristic strategy in the study of folk classification systems is analyzed.

**Results**

The use of 19 biological descriptors (grouped according to 4 salient criteria) and of comparisons among ethnospecies was found. Results suggest the existence of group and specific descriptors. Researchers identified which ethnospecies are considered similar, how less important traits contribute to descriptions, the relation between specific descriptors and ethnospecies, the presence of cognitive prototypes, and the most relevant salient properties from the emic perspective.

**Conclusions**

The estimated importance of attributes descriptors allowed us to identify the spectrum of salient properties relevant from the emic perspective to characterize the stingless bees. In this sense, the analysis proposed here is useful to study folk taxonomy in culturally heterogeneous groups or multicultural regions, where the linguistic elements usually employed cannot be applied.

**Resumen:**

**Antecedentes**

Los seres humanos, al identificar y clasificar a los organismos emplean una combinación de características morfológicas, sensoriales, utilitarias, culturales y ecológicas. Entre los aportes generados desde la etnotaxonomía, se ha obtenido información sobre los caracteres utilizados para identificar y clasificar una gran diversidad de grupos taxonómicos. Sin embargo, aún faltan trabajos donde se comparen los caracteres utilizados en las descripciones de taxones y se analice en qué medida estos descriptores se encuentran representados. Las abejas sin aguijón conforman un diverso grupo de insectos sociales que han sido estudiadas desde la perspectiva etnobiológica, dada su importancia utilitaria y cultural. Los interrogantes que guían este trabajo son ¿Qué elementos tienen en cuenta los pobladores para caracterizar a las abejas sin aguijón? y ¿Qué importancia revisten los mismos en el estudio de las clasificaciones locales?

**Métodos**

Se realizaron entrevistas semiestructuradas y recorridos en áreas rurales con los informantes. Se caracterizan a las etnoespecies conocidas localmente; se identifican los atributos descriptores y los criterios emergentes utilizados para dichas caracterizaciones; y se estima la frecuencia de citas de los atributos descriptores y criterios emergentes. Por otra parte, se comparan los atributos descriptores empleados para cada etnoespecie y se analiza el aporte de las caracterizaciones como estrategia heurística en el estudio de los sistemas de clasificación folclóricos.

**Resultados**

Se halló el empleo de 19 descriptores biológicos (que fueron agrupados en 4 criterios emergentes) y de comparaciones entre etnoespecies. Los resultados sugieren la existencia de descriptores de grupo y descriptores específicos. Se identificaron cuales etnoespecies son consideradas similares, cómo contribuyen a las descripciones los atributos de menor peso, la relación entre los descriptores específicos y las etnoespecies, la presencia de prototipos cognitivos, así como las propiedades emergentes más relevantes desde la perspectiva émica.

**Conclusiones**

La importancia estimada de los atributos descriptores nos ha permitido identificar el espectro de propiedades emergentes que son relevantes desde la perspectiva emic para caracterizar las abejas sin aguijón. En este sentido el análisis aquí propuesto es de utilidad para estudiar taxonomías folclóricas en grupos heterogéneos culturalmente o en regiones pluriculturales, donde los elementos lingüísticos usualmente empleados no son aplicables.

## Background

Human beings employ a combination of morphological, sensorial, utilitarian, cultural and ecological characters when they identify and classify organisms [1]. Morphological characters are the most widely used [2-5] and they constitute the base for classification systems in all societies [2].

Morphological and sensorial characters are those perceived through the senses, that is, they are organoleptic [6]. The first ones are mainly composed of visual stimuli (e.g. color, shape), and the second ones, of touch, auditory and taste perceptions. Utilitarian characters refer to the uses or properties of a resource, while cultural characters involve their aesthetic value, magic or ludic properties [7]. Finally, ecological characters describe habits of species and their relation with the environment [3].

Similarities in the hierarchical structure of folk and scientific classification systems have been reported [2,4,8]. However, it has been proposed that these similarities could be due to influence of the formal academic model of the researcher [7]. Nevertheless, the use of microscopic morphological characters, typical of Linneana taxonomy differs from folk classifications [1,5,7,9].

In academic spheres, the classification and knowledge of organisms and their interactions constitute different disciplines, namely taxonomy and ecology; while for folk cultures these aspects belong to the same knowledge corpus. This corpus is studied by ethnobiology, which analyzes the utilitarian, emotional, and symbolic relations between societies and natural resources [10,11]. Within ethnobiology, ethnotaxonomy studies how different cultures compile, name and classify organisms [4].

Ethnotaxonomy has provided a store of information about the characters that cultures employ when they identify and/or classify a vast diversity of taxonomic groups [1-3,12,13]. Nevertheless, some more research is needed to provide a comparison of the characters employed in the description of taxons, and an analysis of the extent to which those descriptors are represented. These works will allow the evaluation of interrelations among properties of organisms, and ecological and cultural variables (e.g. Bentley and Rodriguez [8]).

The identification of a species implies the recognition of the descriptive traits that, individually or grouped together, allows the observant to associate the organism with a specific category, generally through a name. In the literature, said descriptors are considered elements of either, identification and/or classification, causing inaccuracy of the elements used for identification and classification, respectively [14,15]. That is, perceptive strategies involved in identification (recognition, representation and verification) are not distinguished from logical inferences typical of the classification process (inclusion and contrast) [14]. The present work examines the descriptors used by rural inhabitants from the north of Argentina to characterize Meliponini stingless bees as a first contribution to the definition of the local classification system.

Stingless bees constitute a diverse group of social insects (~391species in America *sensu *Camargo & Pedro [16]) that produce honey, wax, pollen and larvae historically valued by human beings [17,18]. These bees have been studied from an ethnobiological perspective due to their utilitarian and cultural importance in the following communities: Uwa in Colombia [19], Kayapó [3], Pankarare [20], Enawene-Nawe [21], Mby'a in Brazil [22] and Mby'a and Ava Guaraní in Paraguay [23]. In Argentina, in the region we are working at, we have studied the relation of these bees with the Mby'a [24], and among multicultural groups in the border between Brazil and Argentina [25].

The objective of this study is to answer the following questions: What elements do local people from the north of Misiones consider when characterizing stingless bees? and How important are these elements in the study of local classifications? To that end, ethnospecies are characterized according to the perspective of local people. Descriptive traits and salient criteria employed in those characterizations are identified. Frequency of reference of descriptive traits and salient criteria are estimated. Finally, the descriptive traits used in the characterization of the different ethnospecies are compared, and based on this, the contribution of the characterizations, as an heuristic strategy in the study of folk classification systems, is analyzed.

Descriptive traits are those elements employed in the characterization of stingless bees. Although characterization and identification can be synonyms, in the present study they are considered different cognitive processes, each of them associated to a specific methodology. For instance, when the description of an organism is requested, the data obtained will differ whether the specimen is shown or not. In the identification, the interlocutor *identifies *resorting to perception of the stimuli and to memory; whereas in the characterization the interlocutor *characterizes *according to what he remembers resorting just to his memory.

## Methods

### Study area and population

**Figure 1:**
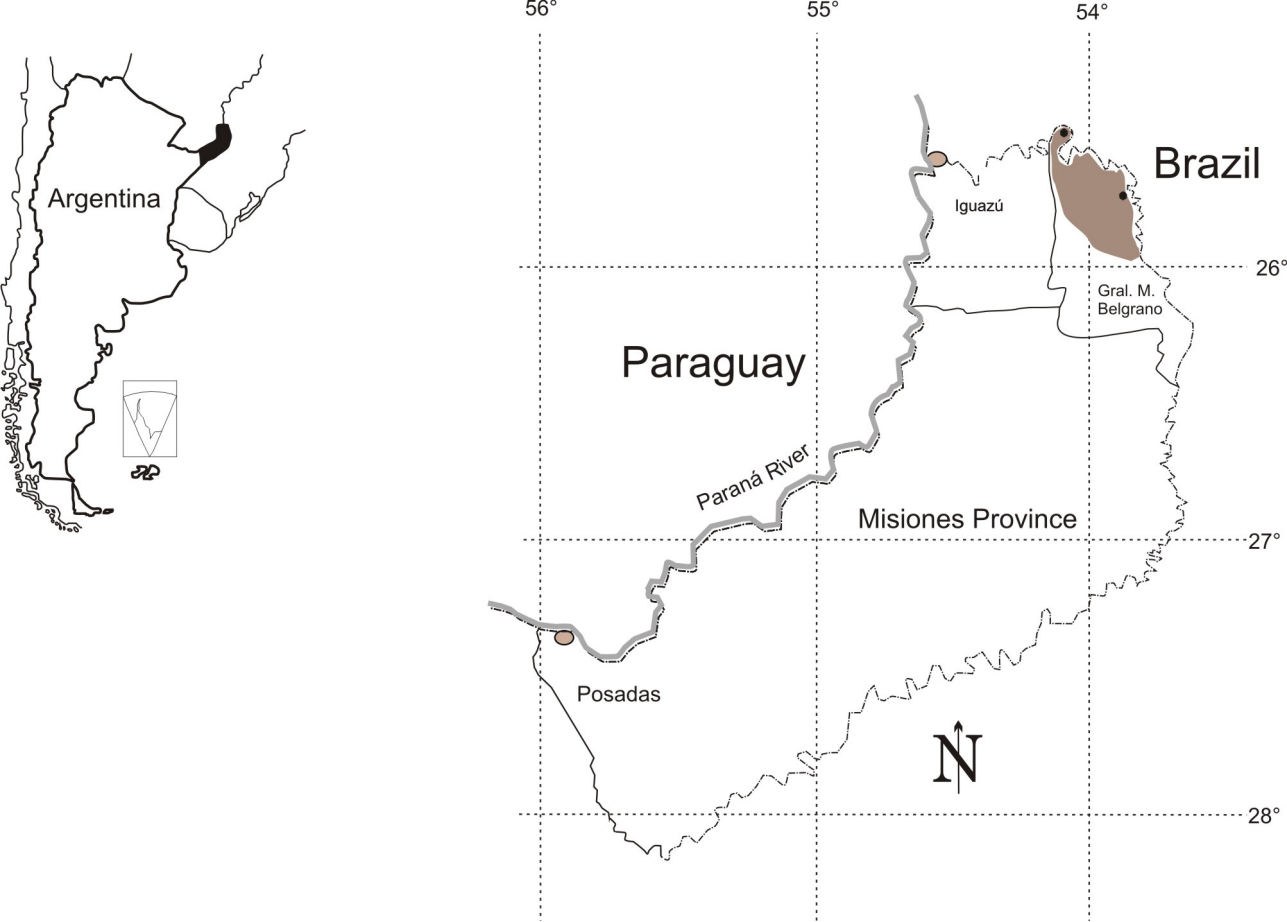
**Studied area**.

The present study was carried out at the north of the province of Misiones (Argentina) in General Manuel Belgrano Department (Figure 1). It borders on Brazil and belongs to the Alto Parana Atlantic Forest; one of the ecoregions of the Atlantic Forest. This is a subtropical semi-deciduous forest with an annual rainfall of 1700-2200 mm, with no marked drought season [26].

The provincial economy is based on primary activities with a limited development of the industrial sector [25,27]. The department has an area of 3276 km^2 ^and a present population of 42,929 inhabitants [28]. From an ethnic point of view, it is characterized by the coexistence of native peoples (Mby'a-Guaraní), European and Asian immigrants who arrived between 1900 and 1940, and Paraguayan and Brazilian families that moved to the province during the 20th century [29].

In the present study we worked with an heterogeneous group referred here to as "criollos" formed by Brazilian and Argentinian populations of German or other European origin and Paraguayan, and Argentinian mestizos. This group presents a regional idiosyncrasy which constitutes their heterogeneous local culture characterized by: a) the influence of the neighboring Brazilian culture (alternatively, Spanish and Portuguese are spoken), b) the lack of self-ascription to a specific ethnic group, and c) the absence of institutions that bring them together as a cultural homogeneous group [25].

### Data collection and analysis

The field work was carried out between July 2007 and December 2009. A total of 65 male rural inhabitants aged 16-79 were interviewed (49.2 years old on average).

Due to the already mentioned characteristics of the population, data collection was carried out in places distant from one another (10-40 km) to gather information as varied as possible about folk knowledge and perceptions of stingless bees.

The selection of informants combined random sampling with the snowball sampling technique [30]. Before each interview, the informed consent of people was obtained, the objectives of the study were informed and the returns of the compiled information were decided. Both methods led to key informants and lay informants (here defined as the ones who knew nine or less, and more than nine ethnospecies, respectively). All of them are small farmers (5-50 ha) who combine tobacco commercial growing with farming in agricultural parcels for their subsistence and for farm animal breeding. The places or colonies where the field work was carried out have minimal infrastructures, dirt roads, insufficient power lines, no running water, and multigrade satellite schools.

During the interviews, informants were asked for descriptions of the mentioned ethnospecies as regards morphology, lifestyles, behaviors, and characters of nests and their entrances ("*piqueras*"). But the complementary application of both semi-directive and free interviews during field work (more than 3 years) dealing with other topics not outlined in this article made it possible to identify other emerging from the responses. Emic characters that best describe the ethnospecies were compiled. Each interviewee was visited twice to six times (all the visits were conducted by FZamudio). Trips around agricultural parcels, forest areas, and rural ways were made accompanied by them. Visits to places where the existence of stingless bee colonies was reported were carried out. Samples were collected and kept in a 70% alcohol solution and later mounted with enthomological pins. They remain stored within the ethnoentomological collection of the research team at the Instituto de Biología Subtropical (IBS-UNAM) (Subtropical Biology Institute). Most of the stingless bees collected were identified by specialists (Dr. Silveira, Dr. C. Rasmussen) and the rest of them were identified according to gender by FZ using Silveira et al.'s keys [31]. Reference specimens of all the ethnospecies were collected, except from *mandasaia *(Cfr. *Melipona quadrifasciata*).

If during the interview individuals belonging to the said species were not observed, descriptions were compared to the descriptions of the collected specimens in this paper, vernacular names are used, so the term "bee" refers to *Apis mellifera*, in order to distinguish among the general terms that make reference to the bees from the Apoidea sub-family.

The descriptions compiled for each ethnospecies are categorized according to the ***Descriptive Traits ***mentioned which in turn were gathered in ***Salient Criteria*. **Both arrangement levels were defined by the researchers, considering the stated *emic *categories. On that basis, the number of times a species was mentioned (N° of references) and the number of times a species was described (N° of reports) were calculated. The importance of descriptive traits was calculated by considering the number of descriptors stated for each criterion (ND) and the frequency of reference (FM), according to the following formula:

$$\begin{array}{c} \text{Frequency of reference FM = }\left[{\text{N}}^{\circ }\text{ of reports}xi/{\text{N}}^{\circ }\text{ of reference to the species}i\right]\times \text{1}00.\;\\ \text{Where }x=\text{descriptive trait};\;i=\text{species described} \end{array}$$

A parametric analysis of variance (ANOVA) was carried out to compare the number of descriptors cited for each criterion (ND) and the frequency of reference of said descriptors (FM) within the salient criteria, meanwhile for the data complied with the suppositions of normality (Shapiro-Wilks *P *= 0.54 and *P *= 0.88, respectively) and homocedasticity (Levene F = 0.28, *P *= 0.8419 and F = 2.08, *P *= 0.12, respectively). Kruskal Wallis' non-parametric method was employed to compare the descriptors according to the FM. Before that, data were converted with natural logarithm (Ln) to reach homoscedasticity of variance (F = 1.36, *P *= 0.1620). Spearman's rank correlation coefficient was employed to calculate independence among the number of reports of each taxon, the number of descriptors cited for each criterion and their frequency of reference (ND and FM respectively).

Finally, the contribution of the characterizations as an heuristic strategy in the study of folk classification systems was analyzed through a Principal Component Analysis (PCA). This analysis allows the study of interdependence of metric variables and the accurate graphic representation of data variability. The main purpose of the technique is reducing data dimension to simplify the problem object of study. Distance between points (in this case ethnospecies) and vectors (in this case descriptors) shows the degree of association, while distance among points shows the degree of similarity. This distance can be obtained through the minimum spanning tree that joins points of observation according to the real distance between them. The angle between trait-vectors indicates the association between them; a 90° angle denotes a null association [32].

For PCA data matrix construction, a consensus index (CI) was calculated according to the following equation: CI = [N° of registers *xi*/N° of informants that describe *i*] × 100 (Molares and Ladio, 2008). Infostat software was used to carry out statistical analyses [32].

## Results

### Stingless bees according to local descriptions

From the 12 ethnospecies mentioned, the most recurrently cited were *yateí, carabozá *and *borá *with 65, 59, and 47 reports respectively. On average, 84% of the informants named and described said ethnospecies, except for *yateí*, which, although the most mentioned, was the only one described in 38.5% of the reports. Within the descriptive strategies used by the respondents to characterize etnoespecies we found both descriptors: biological attributes and comparisons between etnoespecies according to family resemblance between taxa. Among the last ones, some descriptions refer to resemblance or similarity to *yateí *(e.g. "the *tobuna *is like the *yateí *but black"). The descriptive traits cited were grouped into 19 categories, which in turn were grouped into four general salient criteria: organoleptic, ethological and those referred to *piquera *and nest (Table 1). These descriptive traits characterize both the individuals (in their appearance and behavior) and their nests; and they are generally mesoscopic (e.g. shape of wings or spotted pattern) and macroscopic (e.g. shape and size of the bee).

**Table 1:** Salient descriptors used by inhabitants from the north of Misiones to describe and identify stingless bees.

Criteria	Descriptive Traits	Description
**Organoleptic** **(ORG)**	Size	General size or body parts' size *("*bigger than yateí*")*.
	Color	General color, body parts or spotted pattern.
	Texture	Characteristics others than color or shape ("*bright*").
	Shape	Shape traits *("it looks like an ant", "plain abdomen")*.
	Smell	Produced when they attack or are held in hand.

**Ethological** **(ETHO)**	Behavior in the nest	***Docile*: **They do not attack and maintain their activity when a person is close to the nest.
		***Aggressive: ***They attack when a person is close to the nest ("*knocks", "bites", "piss", "causes a burning sensation*").
		***Shy: ***They do not attack or stop their activity (immediate retreat) when a person is close to the nest ("*no bees enter or leave the nest*").
	Forage	***Non-hygienic habits: ***They stand on excrement, urine, dead animals or people's sweat ("*they are filthy*").
		***Cleptoparasitism: ***They steal honey or wax from other stingless bees.
	Defense	Defensive strategies against other insects or non-human enemies *("yateí cut enemies' wings")*.
	Flight	***Flight style or type***: *("it is fast" *or *"it flies like a butterfly")*.
		***Flight sound: ***Sound produced inside the nest ("*they snore like a bee"*) and/or when they fly ("*they buzz"*).

***Piquera*** **(PIQ)**	Materials	Type (resin, mud), hardness ("*iratin *[*piquera*] *melts in the sunlight*"), color and smell.
	Shape	Shape traits ("like a '*charuto' *[cigar]"), or ornamentation designs made from resin in colonies without prominent *piquera *("*it has like rays", "it looks like concreting*").
	Size	Length (short, long), width (thick or thin) and general size.
	Diameter	***Thin: ***Thickness similar to that of bees ("*they enter one at a time"*).
		***Thick: ***Much thicker than the thickness of bees ("*many enter at a time"*).
	Number	More than one type of *piquera*.

**Nest**	Substrate	***Interior (underground): ***Underground nests of variable depth.
		***Interior (holes): ***On trees, constructions or other structures.
		***Exterior: ***External nest placed on branches of trees.
	Shape	Shape traits *("looks like a ball", "like a pot")*.
	Colony size	***Numerous***: Large number of individuals ("*a lot work together"*).
		***Small: ***Small number of individuals ("*just a few"*).
	Materials	Type (wax, mud), hardness, color and smell.

Examples are included (between brackets)

Table 2 shows the number of descriptions obtained for each ethnospecies and the traits synthesized for each species. Within the salient organoleptic criterion, ethnospecies are distinguished following a size pattern from tiny species (*mirí *and *yateí*) to big and bulky species (*guaraipo *and *mandasaia)*. The common honey "Bee" (*A. mellífera) *is usually used to refer to the latest. As regards color, ethnospecies were found to have a single predominant color (*iratín, carabozá, tobuna)*, a spotted pattern on their body or head (*abeja del suelo*) or a striped pattern on the abdomen (*mandasaia, mandurí, guaraipo) *were also found.

**Table 2:** Qualities of descriptive traits used to identify stingless bees ethnospecies and the salient criteria that group said traits

Specie/Vernacular name	Number of Reference	Organoleptic	Ethological	Piquera	Locations of Nests
*Tetragonisca fiebrigi *(Schwarz, 1938)- "yateí"	65	Small, yellow.	Docile. Strong defense.	Tubular piquera, thin (1 cm) and of variable length. It may have more than one.	Inners-trees, walls, other substrates
*Trigona spinipes *(Fabricius, 1793)- "carabozá"	59	Medium-sized. Black.	Aggressive. Non-hygienic.	Generally without piquera. Tubular thick and short entrance, if it has one.	Outer
*Tetragona clavipes *(Fabricius, 1804)- "borá"	47	Medium-sized, yellow, striped abdomen.	Aggressive. Strong defense.	Thick short tubular piquera, though it may not have one.	Inners-trees
*Plebeia spp*. (Schwarz, 1938)- "mirí"	46	Tiny, dark.	Docile/shy. Non-hygienic.	With or without piquera. Described in several ways.	Inners-trees, walls, other substrates
*Schwarziana quadripunctata *(Lepeletier, 1836)- *"*abeja del suelo"	35	Medium-sized, thin, large wings. Black. Light spots on body and head, "bataraz" (grey and white pattern).	Shy. Strong defense.	With tubular piquera or entrance on the ground without piquera (just one hole).	Underground

*Lestrimelitta limao *(Smith, 1863) and/or *L. rufipes *(Friese, 1903)- *"*iratín"	32	Small to medium-sized, thin ("fine"), round head. Black, bright. Strong lemon verbena or citronella smell.	Docile/shy Robber bees "snore" inside the nest	Tubular piquera with 2 shapes; a) large (10-15 cm) and wide (3-4 cm); b) a wide main piquera surrounded by numerous blind wax tubes.	Inners-trees
*Melipona quadrifasciata *(Lepeletier, 1836*)- *"mandasaia"	27	Big, strong. Mainly yellow or black body. Conspicuous. Stripped abdomen.	Shy	Without piquera. Just one bee-diametered hole.	Inners-trees
*Scaptotrigona depilis *(Moure, 1942) and/or *S. bipunctata *(Lepeletier, 1836)- "tobuna"	24	Medium-sized with short body or retracted abdomen. Black.	Aggressive. Strong defense.	Straight tubular piquera, thick (2-4 cm) and to 12 cm long.	Inners-trees
*Melipona obscurior *(Moure, 1971)- "mandurí"	24	Medium to big. Black, brown or yellowish. White face. Visible striped abdomen. "bataraz" or "tobiana" (black and white patterns).	Shy.	Without piquera. Just one bee-diametered hole covered in wax. It may have ornamentations.	Inners-trees

*Melipona bicolor *(Lepeletier, 1836)- "guaraipo"	22	Big, strong. Mainly black. Hairy. Striped abdomen, "barcina" (with black and brown stripes).	Shy. "buzz" like a bee.	Without piquera. Just one bee-diametered hole covered in wax. It may have ornamentations.	Inners-trees
*Cephalotrigona capitata *(Smith, 1854)- "mambuca"	13	Medium to big. Black or brown. Big head.	Shy. "snore" in the nest.	Without piquera. Just one bee-diametered hole covered in wax.	Inners-trees
*Oxitrigona tataira *(Smith, 1863)- "cagafuego"	4	Medium to small. Brown or yellow. "Ant head".	Aggressive (burning sensation)	With or without tubular piquera.	Inners-trees

According to their behavior (ethological criterion), bees are characterized as docile, like *yateí *and *mirí*, shy or aggressive. *Mandurí, mandasaia, guaraipo *and *mambuca *are grouped within shy bees, as they show similar dissuasive defensive strategies. It was mentioned that they are "wary" bees as when they hear some noise, they stop their activity, that is, "no bees enter or leave the nest". Shy behavior of these species coincides with absence of tubular *piqueras *in their nests, which makes them less conspicuous and difficult to detect (Table 2). On the other hand aggressive bees are those which attack in large groups biting hair and clothes; e.g. *carabozá *(also known as *corta pelo*- hair cutter-), *borá, tobuna *and *cagafuego*. According to the reports, *carabozá *and *borá *also have a substance or "glue" on their hind legs that causes a burning sensation on the skin. C*agafuego *bees differ from the others in that during the attack they release a substance which produces sores on the skin.

As regards *piqueras*, they exhibit different shapes and sizes, even within the same ethnospecies. Ethnospecies are grouped according to the absence of *piquera*, the presence of one *piquera *(thick or thin) or multiple *piqueras *(Table 2).

As regards nests, collected data shows that most of the species cited nest in holes in tree trunks, or less frequently, in other types of holes like crevices within rocks or wall holes. *Carabozá *nests in external nests, generally on top of trees, which makes them easily visible. Its nest is described as a "mud ball" and is compared to "*lechiguanas*' nests" (*Brachygastra *sp. and *Polybia *sp. wasp species), "*termites*' nest" (Isoptera order) or "*hornero*'s nest" (*Furnarius rufus *passerine bird). The *abeja del suelo *-ground bee-, as its name indicates, nests underground. Its nest is described as a "pot" or "urn" built with a hard material commonly referred to as "shell" or "bark".

Finally, we identified descriptive traits exclusively cited for one or two ethnospecies. Among them, we can mention: the food "stealing" behavior of *iratín *bees (cleptoparasitism), the *cedrón *(*Aloysia citriodora, Cymbopogon *sp.) and *citronella *(*C. citratus) *smell of *iratín *and *borá*, and the *carabozá's *and *mirí's *forage habits considered slightly non-hygienic.

### Representative descriptive traits of stingless bees

The descriptive traits mostly cited by interviewees refer to organoleptic characteristics of the individuals and to their behavior, followed by those describing the entrance to the nest and the nest itself (Table 2).

When the criteria according to the frequency of reference and to the number of descriptors were compared, it was found that in both, Organoleptic and *Piquera *had the highest values. The average frequency of reference to the Organoleptic criterion is significantly higher than the reference to Nest and Ethological criteria. And the average number of descriptors cited for *Piquera *criterion is significantly higher than the average number of descriptors cited for Nest and Ethological criteria (Table 3).

**Table 3:** Frequency of reference (FM) and average number of descriptors (ND) used by inhabitants from the north of Misiones to characterize stingless bees.

	Nest	Ethological	Organoleptic	*Piquera*	F	P
FM	44.4 (c)	72.7 (bc)	112.1 (a)	75.5 (ba)	5.9	0.0018
Range	18.8-112.2	40.6-105	12-200	20-174.2		
S.D.	31.61	21.47	49.4	49.5		

ND	2.17 (c)	2.5 (bc)	3.3 (ba)	4 (a)	10.2	< 0.0001
Range	1-5	1-4	1-5	1-5		
S.D.	0.9	0.8	0.9	0.9		

Descriptors grouped into the following criteria are compared (ANOVA): Nest (N = 12), Ethological (N = 12), Organoleptic (N = 12), and *Piquera *(N = 11). Different letters indicate significant differences among variables (alpha 0.05)

A positive correlation was observed between the number of ethnospecies described per informant and the frequency of reference to descriptive traits (R2 = 0.86, *P *= 3.20E-04). No correlation was found between the frequency of reference to said descriptive traits and the average number of the descriptors cited (R2 = -0.3 *P *= 0.54). This indicates that the frequency of reference to each trait is independent from the number of descriptions reported for each ethnospecies.

**Figure 2:**
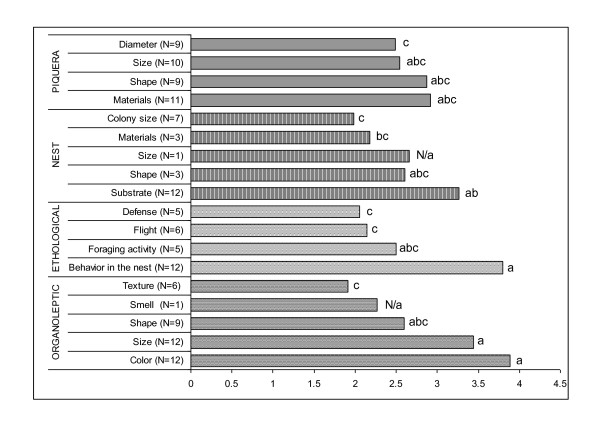
**Natural logarithm of the frequency of reference to descriptive traits used in descriptions of stingless bees ethnospecies**. Different letters indicate significant differences among descriptors (Kruskal Wallis, alpha 0.05). N/a: Not applicable.

The data collected show that Color, Size and Behavior in nest and Nesting substrate constitute the most cited descriptors with a similar frequency of reference. However, no important difference was found among those descriptors and most of the less frequently mentioned descriptive traits, except for Flight, Defense, Size of colony, Texture of bees, Number of *piqueras *of the colonies, and Nest material (although the last one does not differ from Nesting substrate) (Figure 2).

Finally, since descriptors referred to Nest size and Smell of bees were cited only once and only for one ethnospecies (*carabozá *and *iratín*, respectively), they were not considered for the comparisons.

### How are ethnospecies grouped according to descriptors

**Figure 3:**
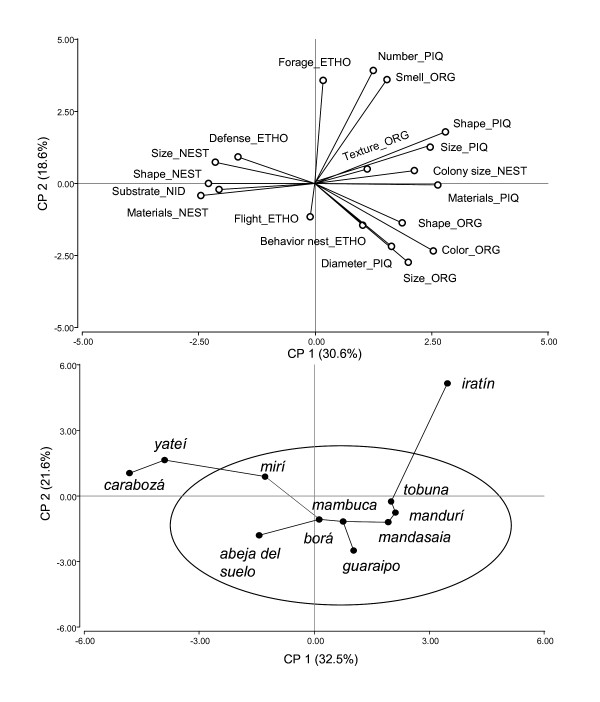
**Graphic representation of Principal Component Analysis (PCA)**. Figures a and b are part of one graph but they were separated for a better reading. a) Distribution of descriptors (components) according to axis 1 and 2, b) Distribution of ethnospecies with minimal spanning tree. Abbreviations, (descriptor_criterion): ORG = organoleptic; PIQ = piquera; ETO = ethological; Defense = defense against non-human enemies; Materials = materials for building (*piquera *or nest); Number = number of *piqueras*, Substrate = nest substrate.

When analyzing the interdependence between descriptors used for the characterization of ethnospecies, it was found that the first three components of the Principal Component Analysis explain 65% of stored variation of the data. According to the distribution of descriptive traits, Component 1 (PC1) separates ethnospecies characterized with Nest descriptors from those characterized with *Piquera *and Organoleptic traits (Figure 3). Consequently, it is inferred that the ethnospecies *carabozá *and *yateí *are conceptually different from the rest as regards these elements (Figure 3a, b). Then, *carabozá *is the only ethnospecies that has an external large nest (Table 2). While *yateí *differs from the rest in its defensive behavior when facing attacks from other organisms (Table 2). According to the minimum spanning tree (Figure 3c), *mirí *and *abeja del suelo *ethnospecies are closer to *borá *than to *yateí*.

Though, in general, ethological descriptive traits do not contribute to the conformation of Component 1, individually, they are associated to some ethnospecies on which there is a greater consensus to this respect. For instance, Flight descriptor refers to the flight activity of *abeja del suelo *or to the sounds produced inside the nest by *mambuca*, or during flight by *guaraipo *(Figure 3b).

Component 2 (PC2), on the other hand, separates best described ethnospecies according to Size, Color and Diameter of the *piquera *from Forage, Smell, and Number of *piqueras *descriptors. Those species grouped at the ellipse of Figure 3 differ from *iratín*, an ethnospecies that releases a strong smell when captured or when attacking other nests, has a large piquera and is considered a *"*robber bee*"*.

## Discussion and conclusion

Results reflect the use of a great variety of descriptors which coincides with the information collected in other regions [5,9]. This may be related to the stingless bee regional richness (~16 species) [33], and to their ecological diversity, as well as to local knowledge variability in the studied population.

Firstly utility and cultural descriptors were not identified unlike results obtained in other cultures [3,5,7]. Although this may be linked to the use of semi-directive interviews, information not contained in this work shows that utilitarian descriptors are referred to while expressing contrasts within the classification process as opposed to descriptors which are used to characterize etnoespecies. These results are explained from the difference between the description and classification processes noticed in this study. On the other hand the ambiguity of criteria according to the context of use and to the interpretation of the researchers should also be considered. For example an etnoespecies aggressive behavior apart from being an ecological descriptor can be useful for people when planning the honey harvest.

Organoleptic criterion gathers most cited descriptors, similarly to what was mentioned by Newmaster et al. [1,5]. In addition, the most cited descriptors in the present study; namely color and size, constitute the most used descriptors for other animal groups [3,13,20,34,35]. Therefore, they can be considered universal descriptors. These descriptors are commonly used in formal taxonomy [31]. However, the ecological descriptors related to the behavior of stingless bees are salient elements in local descriptions of the taxa resembling those observed between Kayapo and Mby'a for the same group of insects [3,22,24].

The results of the quantification of descriptors together with those of the Principal Component Analysis suggest the existence of two types of descriptors: group and specific descriptors, also called additional characters [12,34]. The use of this analytical tool allows the visualization of similarities among ethnospecies, of the participation of less important traits, and also of the relation between specific descriptors and ethnospecies.

In fact, the groupings observed in Figure 3 are explained by universal and group descriptors. Within them, there are descriptors which are more frequently used to describe stingless bees as a whole (e.g. Organoleptic, *Piquera*) (they show a greater eigenvalue in the PCA) and others, such as nest behavior and nest substrate. Besides, in the same figure, location of ethnospecies not included within the main group contained in the ellipse (*iratín, carabozá *and *yateí) *could be explained considering their outstanding and exclusive characteristics, that is, specific descriptors such as nest entrance and cleptobiotic behavior of *iratín*, external nest and aggressive behavior of *carabozá*, and the defensive behavior of *yateí *when it is attacked by other insects [36].

However, the location of *yateí *interpreted in a wider context suggests that it could be a prototypical species which should be further studied. According to Rosch [37], informal groupings are made for a prototypical specimen that best expresses the traits that define a given domain, and that constitutes a reference cognitive point in comparisons to the type "X is like Y". This could explain why *yateí*, the most cited species, has been one of the less described, although it is the most used to describe other species through comparisons. At the same time, *yateí *is the only stingless bee species bred in the region and commonly used for food, medicine, and aesthetic-recreational purposes [25].

In short, the estimated importance of descriptive traits has allowed us to identify the spectrum of salient properties that are relevant, from the *emic *perspective, to characterize stingless bees. The additional use of quantitative and exploratory techniques has allowed us to identify conceptual characterization patterns, which should be further studied after the analysis of folk classification systems. In this sense, the analysis proposed here is useful as an introduction to further study folk taxonomies in culturally heterogeneous groups or in multicultural regions where the usually employed lexical elements [4] are not applicable.

## Competing interests

The authors declare that they have no competing interests.

## Authors' contributions

FZ carried out the field work and the statistical analysis of the information. Both FZ and NH participated in the planning, design of this study, the analysis of the information, wrote the manuscript and read and approved the final manuscript.

## Authors' information

Fernando Zamudio. Biologist, Universidad Nacional de Córdoba, UNC (National University of Cordoba). Argentina. M.S. degree in Natural Resources and Rural Development, ECOSUR. México. Doctoral Student, UNC. CONICET. Centro de Investigaciones del Bosque Atlántico e Instituto de Biología Subtropical, (Atlantic Forest Investigation Center and Subtropical Biology Institute), Universidad Nacional de Misiones (National University of Misiones) (CeIBA, IBS-UNAM), Andresito 27, CP 3370, Puerto Iguazú, Misiones, Argentina. E-mail: zamufer@yahoo.com.ar.

Norma I. Hilgert. Biologist and Doctor of Science, UNC. Associate Researcher at CONICET. Professor at the School of Forest Science, IBS-UNAM, CeIBA. E-mail: normahilgert@yahoo.com.ar.
